# Marked increase in community-associated methicillin-resistant *Staphylococcus aureus* infections, Western Australia, 2004–2018

**DOI:** 10.1017/S0950268820000849

**Published:** 2020-04-23

**Authors:** L. E. Bloomfield, G. W. Coombs, S. Tempone, P. K. Armstrong

**Affiliations:** 1 Communicable Disease Control Directorate, Perth, Western Australia; 2 Edith Cowan University, Perth, Western Australia; 3 The University of Notre Dame, Fremantle, Western Australia; 4 PathWest Laboratory Medicine, Perth, Western Australia; 5 Murdoch University, Perth, Western Australia

**Keywords:** Community outbreaks, emerging infections, methicillin – *S. aureus* resistant to (MRSA)

## Abstract

This study presents enhanced surveillance data from 2004 to 2018 for all community-associated methicillin-resistant *Staphylococcus aureus* (CA-MRSA) specimens collected in Western Australia (WA), and describes the changing epidemiology over this period. A total of 57 557 cases were reviewed. Annual incidence rates increased from 86.2 cases per 100 000 population to 245.6 per 100 000 population (IRR = 2.9, CI_95_ 2.7–3.0). The proportion of isolates carrying Panton–Valentine leucocidin (PVL)-associated genes increased from 3.4% to 59.8% (*χ*^2^ test for trend 7021.9, *P* < 0.001). The emergence of PVL-positive, ‘Queensland CA-MRSA’ (ST93-IV) and ‘WA 121’ (ST5-IV) accounted for the majority of increases in CA-MRSA across the study period. It is unclear why some clones are more prolific in certain regions. In WA, CA-MRSA rates increase as indices of temperature and humidity increase after controlling for socioeconomic disadvantage. We suggest climatic conditions may contribute to transmission, along with other socio-behavioural factors. A better understanding of the ability for certain clones to form ecological niches and cause outbreaks is required.

## Background

*Staphylococcus aureus* is the most commonly isolated bacterial pathogen in humans and is an important cause of skin and soft-tissue infections (SSTIs), pneumonia, septic arthritis, endocarditis, osteomyelitis, foreign-body infections and sepsis [1]. Methicillin-resistant *S. aureus* (MRSA) isolates are resistant to penicillins and other *β*-lactam antibiotics and in the past were confined mainly to health care environments [2]. However, over the last 30 years, there has been an increase in the number of MRSA infections in persons without the usual healthcare-associated risk factors. An increased recognition of new MRSA clones, commonly referred to as community-associated MRSA (CA-MRSA) in order to distinguish them from their healthcare-associated counterparts, is responsible for such infections [2].

In Australia, CA-MRSA infections first emerged from the northern Kimberley region in Western Australia (WA) in the early 1990s, mainly associated with Aboriginal people living in remote communities [3]. Since that time, the heavy burden of staphylococcal disease and an increasing prevalence of CA-MRSA in these populations across northern Australia have been noted [4].

Different CA-MRSA strains have been demonstrated to have found ecological niches, with different regions experiencing outbreaks largely comprising different dominant strains [1], such as USA300 in North America, ST80-IV ‘European clone’ in countries of the Gulf Cooperation Council [5], the ST30-IV ‘South Western Pacific clone’ in New Zealand [2]. While the global emergence and spread of CA-MRSA have previously been well-documented [6], Australia has been no exception [7], with ST93-IV ‘Queensland CA-MRSA’ noted as the predominant clone in Australia for over a decade [8].

Western Australia has adopted an aggressive approach to MRSA management, with stringent policy requirements for the screening, reporting and management of cases introduced in the 1980s [9]. Colonisation or infection with MRSA has been a notifiable condition in WA since 1982, and since 1997 all MRSA isolates have been referred to the PathWest Gram-positive typing Laboratory (GPTL) where isolates are characterised as either healthcare-associated MRSA (HA-MRSA) or CA-MRSA based on molecular markers [10].

Since 2003, GPTL has employed multilocus sequence typing (MLST) and staphylococcal chromosomal cassette *mec* (SCC*mec*) typing which has enabled the identification of novel clones not previously reported. This study presents 15 years of enhanced surveillance data from specimens collected in WA, and describes the changing epidemiology and burden of disease over this period.

## CA-MRSA and HA-MRSA

Phenotypically, CA-MRSA clones are generally resistant to fewer non-*β*-lactam antibiotics than HA-MRSA clones [11]. Clinically, CA-MRSA infections generally occur in younger, otherwise healthy patients and are associated predominantly with SSTIs, but may also cause severe clinical syndromes such as necrotizing pneumonia and sepsis [1, 12]

In WA, CA-MRSA clones are frequently responsible for infections detected in the hospital setting, including those classified as nosocomial infections [13]. Consequently, classification of CA-MRSA and HA-MRSA clones based solely on hospitalisation and risk factors for healthcare may lead to frequent differential misclassification [11] with molecular typing methods considered a more accurate means of identifying and classifying CA-MRSA clones.

CA-MRSA may be differentiated from HA-MRSA clones by genotypic features such as MLST, having a relatively smaller SCC*mec* elements (typically type IV and V), and by the frequent presence of the Panton–Valentine leucocidin (PVL) *lukF-PV* and *lukS-PV* associated genes [11]. Typing data from GPTL are used to direct patient management and appropriate infection control responses within WA healthcare facilities.

### PVL status

The protein coded by the *lukF-PV* and *lukS-PV* PVL-associated genes mediates leukocyte and tissue destruction, and it has been suggested that its presence may cause more severe SSTIs than PVL-negative (PVL−) isolates [12, 14]. However, controversy remains regarding whether observed increases in disease severity are due to the presence of the PVL toxin, or if it is a marker for other virulence factors [1]. Conflicting evidence from animal and human studies has led to inconclusive findings of the exact role of PVL in disease severity; however, evidence suggests that the effects may be strain-specific [1].

Although PVL-positive (PVL+) isolates do not appear to be related to increases in bacteraemia or mortality [12], a demonstrated propensity to cause more invasive SSTIs would place a significant burden on healthcare services if the incidence of these strains increased, with more complex procedures required to treat and manage these infections.

## Methods

### Genotyping

In WA, the classification of CA-MRSA and HA-MRSA clones is determined by a combination of the molecular analysis of the seven housekeeping genes sequence types using MLST, and the SCC*mec* type using multiplex PCR [15]. Prior to 2014, all CA-MRSA (regardless of PVL status) wereindividually typed. Increases in case numbers and subsequent workload, combined with the low applicability of the results to patient management, led to a decision to group all PVL− CA-MRSA under one typing result. This classification was applied retrospectively to all data, and therefore all PVL− CA-MRSA are grouped together. Typing data for PVL+ isolates are available for the full study period.

### Epidemiology

Cases were extracted with the following inclusion criteria:

- Collection date between 1 January 2004 and 31 December 2018
- Persons with a WA address (classified by residential postcode) at the time a sample was collected
- Sample was positive for a CA-MRSA clone identified by GPTL
- Sample was identified as a clinical specimen (screening specimens excluded)
- Specimen was not a duplicate (specimen of the same strain collected from the same patient)

Estimated resident population (ERP) figures from 2004 to 2017 were obtained from the Australian Bureau of Statistics for each region. Regional ERP for 2018 was estimated based on linear population growth trends for the period 2015–2017.

### Study population

WA comprises approximately 2.6 million km^2^ and is commonly divided into eight major geographical regions. Each of these regions varies widely on a number of factors, including geography and climate, population density, proportion of Aboriginal residents, and indices of rurality and remoteness. Regional areas of the state, particularly northern and easterly regions, contain populations with some of the highest indices of socio-economic disadvantage in Australia [16]. The population of WA is approximately 2.6 million people, with almost three-quarters of the population residing in metropolitan Perth [17].

Regions were further categorised based on their climatic properties, using Bureau of Meteorology data for climate zones based on temperature and humidity [18].

**Fig. 1.**
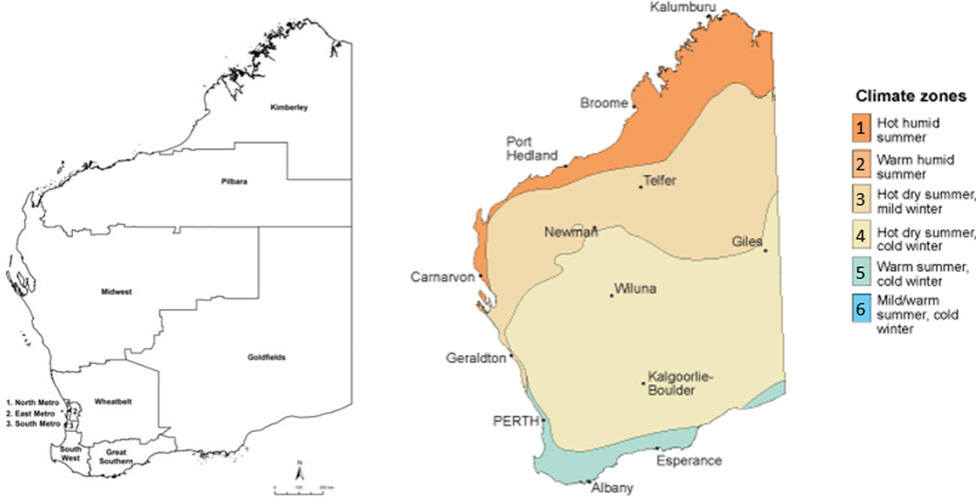
Regions and climatic zones, Western Australia, adapted from Bureau of Meteorology [18].

Regions were assigned to climate zones based on having a majority of regional landmass and/or population within a particular band. Zone 1 (hot, humid summer) contained the northernmost Kimberley region, Zone 3 (hot dry summer, mild winter) contained the northern-central Pilbara and Midwest regions, Zone 4 (hot dry summer, cold winter) contained the central Wheatbelt and Goldfields regions, and Zone 5 (warm summer, cold winter) contained the southerly Metropolitan, Southwest and Great Southern regions (Fig. 1). Deciles taken from the ABS Indices of Relative Socio-Economic Advantage and Disadvantage (IRSAD) were applied based on patient postcode.

### Statistical analysis

Data analysis was performed using R version 3.5.2 (R Foundation for Statistical Computing; Vienna, Austria). Annual incidence rates per 100 000 population were calculated. A Wilcoxon rank-sum test was used to assess the difference in continuous variables. Multivariable logistic regression was used to assess age, sex and climatic region of residence as independent risk factors for PVL+ CA-MRSA. Cases with missing data for sex and age group were deleted listwise. Negative binomial regression models were selected due to overdispersion of annual count data, and were used to estimate year-adjusted regional incidence rate ratios (IRRs) and associated 95% confidence intervals (CI_95_).

## Results

A total of 57 557 cases of CA-MRSA reported to GPTL between 1 January 2004 and 31 December 2018 were extracted from the database in January 2019. While overall, the majority (57%) of cases reported over the 15-year period were PVL− CA-MRSA, assessment of annual trends demonstrates the proportion of PVL+ CA-MRSA increased from 3.4% to 59.8%, comprising the majority of isolates since 2015 (*χ*^2^ test for trend 7021.9, *P* < 0.0001) (Table 1).

**Table 1.** CA-MRSA cases, WA, 2004–2018

	PVL negative CA-MRSA	PVL positive CA-MRSA	Total
	*n* (%)	*n* (%)	*N*
Total	32 868 (57.6%)	24 689 (42.4%)	57 557
Sex			
Female	17 263 (60.4%)	11 464 (39.6%)	28 727
Male	15 540 (54.7%)	13 205 (45.3%)	28 745
Unknown	65 (76.5%)	20 (23.5%)	85
Year			
2004	1649 (96.7%)	58 (3.3%)	1707
2005	1573 (94.1%)	101 (5.9%)	1674
2006	1693 (92%)	153 (8%)	1846
2007	1718 (83.8%)	356 (16.2%)	2074
2008	1916 (78.9%)	540 (21.1%)	2456
2009	2077 (75.8%)	712 (24.2%)	2789
2010	1933 (71.7%)	811 (28.3%)	2744
2011	2289 (67.6%)	1156 (32.4%)	3445
2012	2239 (62.5%)	1450 (37.5%)	3689
2013	2364 (56.1%)	2006 (43.9%)	4370
2014	2675 (51.8%)	2511 (48.2%)	5186
2015	2769 (46.2%)	3157 (53.8%)	5926
2016	2644 (42%)	3698 (58%)	6342
2017	2802 (40.3%)	4220 (59.7%)	7022
2018	2527 (41.2%)	3760 (58.8%)	6287
Age group			
0–9 years	3891 (41.1%)	5380 (58.9%)	9271
10–19 years	2306 (34.9%)	4211 (65.1%)	6517
20–29 years	3025 (40%)	4442 (60%)	7467
30–39 years	3272 (45.4%)	3898 (54.6%)	7170
40–49 years	3145 (51.4%)	3013 (48.6%)	6158
50–59 years	3041 (61.6%)	1955 (38.4%)	4996
60–69 years	3225 (76.1%)	1024 (23.9%)	4249
70–79 years	3475 (89.4%)	431 (10.6%)	3906
80+ years	7475 (95.9%)	334 (4.1%)	8970
Unknown	13 (93.3%)	1 (6.7%)	14
Climate zone			
Zone 1	2570 (28.4%)	6402 (71.6%)	8972
Zone 3	3579 (45.9%)	4264 (54.1%)	7843
Zone 4	2187 (55.8%)	1796 (44.2%)	3983
Zone 5	24 532 (67.4%)	12 227 (32.6%)	36 759

### Incidence rates by climatic region and PVL status

**Fig. 2.**
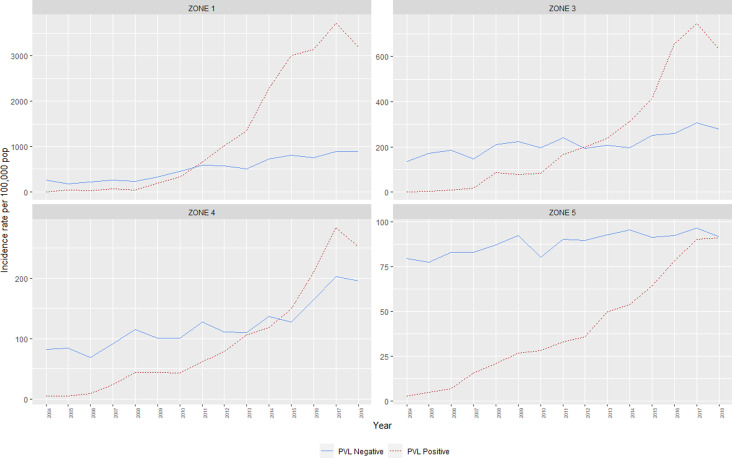
Incidence rates, CA-MRSA, Western Australia, 2004–2018.

Between 2004 and 2018, annual incidence rates of CA-MRSA in WA increased almost threefold, from 86.2 cases per 100 000 population to 245.6 per 100 000 population (IRR = 2.9, CI_95_ 2.7–3.0). At the commencement of the monitoring period, there were some differences noted in the incidence rates of CA-MRSA in the hotter climatic zones, with significantly higher total rates in Zone 1 (IRR = 3.2, CI_95_ 2.5–3.9) and Zone 3 (IRR = 1.7, CI_95_ 1.4–2.0) compared with the cooler Zone 5 (reference category) in the south of the state. The differences noted at this time are entirely attributable to PVL− strains, with very few cases of PVL+ CA-MRSA (*n* = 58) reported within the state in 2004 (Fig. 2).

The IRR of all CA-MRSA increased significantly as indices of temperature and humidity increased across climatic zones (Table 2). After adjusting for year in the negative binomial model, a trend was identified for both PVL− and PVL+ CA-MRSA. Using Zone 5 (warm summer, cold winter) as a reference, the IRR for all CA-MRSA was over ninefold higher in total in the northerly region of the state (Zone 1), compared to the reference (Zone 5).

**Table 2.** Year-adjusted incidence rate ratios, by climate zone

	PVL^−^ CA-MRSA			PVL^+^ CA-MRSA			All CA-MRSA		
Climate zone	IRR^a^	CI_95_		IRR^a^	CI_95_		IRR^a^	CI_95_	
Zone 1	5.38	4.70	6.15	15.74	11.70	21.16	9.11	7.22	11.49
Zone 3	2.37	2.08	2.70	3.49	2.60	4.68	2.90	2.31	3.63
Zone 4	1.30	1.13	1.48	1.74	1.29	2.34	1.41	1.12	1.77
Zone 5	1.00	Ref		1.00	Ref		1.00	Ref	

a Adjusted for year of specimen collection.

The median age for PVL+ cases was significantly lower at 26.7 years, compared with 54.5 years for PVL− cases (*P* < 0.001). Multivariable logistic regression adjusting for collection year and IRSAD demonstrated that male sex, younger age, and residence in hotter, more humid regions were positively associated with infection with PVL+ CA-MRSA, compared to other strains (Table 3).

**Table 3.** Factors associated with a PVL+ CA-MRSA infection

	aOR^a^	CI_95_	
Sex			
Female	1.00	Ref	
Male	1.27	1.22	1.32
Climate zone			
Zone 1	2.24	2.07	2.41
Zone 3	1.33	1.25	1.42
Zone 4	1.10	1.02	1.20
Zone 5	1.00	Ref	
Age group			
0–9 years	20.20	17.92	22.84
10–19 years	31.22	27.60	35.43
20–29 years	25.05	22.19	28.36
30–39 years	19.76	17.50	22.37
40–49 years	15.13	13.38	17.16
50–59 years	10.80	9.52	12.28
60–69 years	5.52	4.84	6.32
70–79 years	2.46	2.12	2.87
80+ years	1.00	Ref	

a Adjusted for age group, sex, climate zone, IRSAD and year.

### Molecular biology

**Fig. 3.**
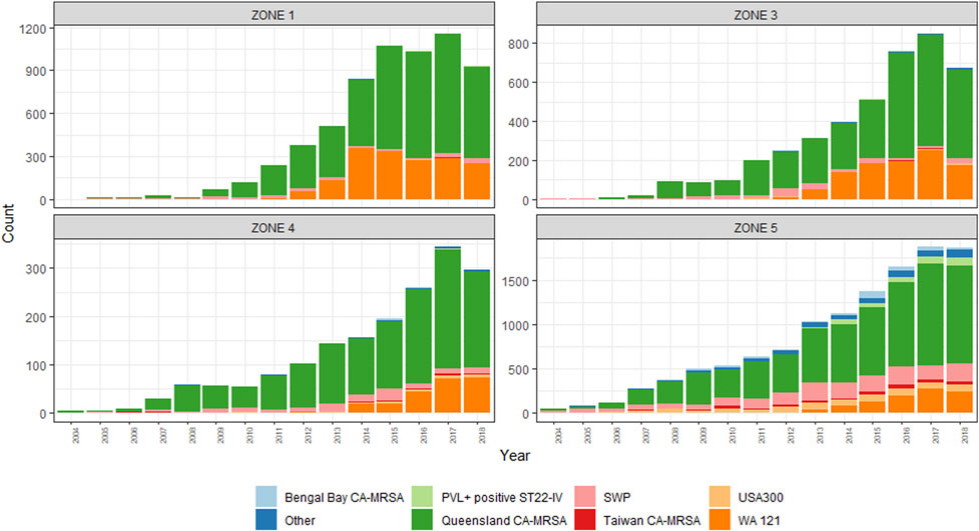
PVL-positive CA-MRSA clones, by climatic region, 2004–2018.

The emergence of two PVL+ clones, ‘Queensland CA-MRSA’ (ST93-IV) and ‘WA121’ (ST5-IV), account for the majority of increases in CA-MRSA across the study period. Proportionally, Queensland CA-MRSA accounted for 1.6% of all CA-MRSA strains in 2004; this increased to 37.9% of the total by 2018. Case numbers for this clone increased across the state, and is now the single most prolific strain in WA. As shown in Figure 3, the case numbers of this clone are disproportionately higher in Zone 1, which has comparable counts to Zone 5, despite Zone 5 having a population approximately 66 times the size.

The emergence of WA121 occurred in 2011 with five cases of this previously undetected clone occurring in this year. This number increased rapidly to 73 cases in 2012. Since this time, year-on-year increases in WA121 have been observed, in 2018 accounted for 740/6287 (11.8%) of all reported CA-MRSA. The same regions in which Queensland CA-MRSA has proliferated again account the majority of increase in case numbers of WA121.

Greater diversity among PVL+ isolates is noted in the southerly, cooler, more densely populated regions, with South West Pacific (SWP) CA-MRSA (ST30-IV) and the PVL+ ST22-IV representing a sizeable minority within these zones. While PVL− CA-MRSA rates increased overall during this period, proportionally PVL− isolates declined from 97% of the total to 40%. This demonstrates that rather than replacement, PVL+ isolates have had an additive effect on the over rates of CA-MRSA in WA over the last 15 years.

## Discussion

This study reports a sustained increase in clinical CA-MRSA rates in WA, with an apparent commencement of an outbreak of PVL+ MRSA in regional areas commencing 2009. Reports of increases in CA-MRSA are occurring globally, including Europe, the Asia-Pacific region and North America [1, 19, 20]. The introduction and proliferation of Queensland CA-MRSA in the last 15 years have dominated the landscape of CA-MRSA in WA, with this clone alone accounting for almost 40% of all CA-MRSA cases in the most recent reporting period.

In addition, the recent emergence of WA121 appears to follow a similar pattern as the Queensland CA-MRSA, with a sudden emergence and rapid proliferation in remote communities. Given the demonstrated ability for a newly introduced clone to add to the burden of disease in the community, the emergence of a new PVL+ clone in these regions is of particular concern. This same clone rapidly emerged in New Zealand between 2006 and 2011 [21] to become a dominant strain in that country during this period.

The emergence and spread of CA-MRSA, particularly in the Kimberley region, have previously been documented [9, 22]. The precise reason for the emergence and spread of PVL+ strains during this period is not currently known; however, a disproportionate burden of PVL+ CA-MRSA among younger, Aboriginal Australians is well established [7, 23–25]. Reasons for the increased number of cases within these populations are complex, and centre around environmental and behavioural determinants, such as domestic overcrowding, comorbidities (especially the presence of other skin conditions), hygiene and antibiotic use [23, 24]. Although Aboriginal status was not available as part of the dataset, the increase in case numbers associated with regions and remote areas with a high proportion of Aboriginal residents [26] suggests that Aboriginal people are likely over-represented in these data.

Queensland CA-MRSA has previously been implicated as a major contributor to the burden of disease in Aboriginal populations, suggesting particular genetic factors unique to this clone may have facilitated its spread in this population across Australia [24, 27]. The epidemiology of PVL+ USA300 within North America mirrors the WA experience with Queensland CA-MRSA in many ways – a rapid emergence and subsequent dominance of a particular well-adapted clone [1], which is now responsible for an increasing number of healthcare-associated infections [28].

Despite numerous incursions of USA300 observed over the surveillance period, this strain has not proliferated in WA. Similarly, despite small-scale outbreaks of Queensland CA-MRSA in the UK [29], it has not resulted in the same widespread dissemination in this country [1]. In fact, this clone has had apparently very limited spread, even within the Asia-Pacific region [19]. Reasons why some strains seem to dominate in certain regions and not others remain unclear; however, this suggests that there are other factors, outside of a susceptible population and apparently fit strain, which allow establishment and transmission in a certain region.

The rate of PVL+ CA-MRSA in the highly populous Zone 5 has increased, but is still lower than that of PVL− CA-MRSA. Rates of CA-MRSA in other regions that also contain areas of high disadvantage have not increased as markedly as within the state's north. This suggests that a combination of socio-behavioural and environmental factors may be reducing the ability of PVL+ CA-MRSA to proliferate within more temperate geographic locations within the state.

Climatic influences on MRSA SSTIs have previously been explored [30], although a paucity of evidence exists. The data collected by Sahoo *et al.* suggest a combination of temperature and humidity; namely, average weekly maximum temperatures above 33 °C and an average weekly relative humidity between 55% and 78% was associated with an increase in *S. aureus*-associated SSTIs [30]. Average maximum temperature and humidity data across climate bands published by the Bureau of Meteorology suggest that optimum conditions for colonisation and spread of MRSA are present in northerly climate zones of the state for a large proportion of the year.

Further work linking transmission to climatic factors is recommended to better describe this phenomenon. Factors outside of the presence of PVL-associated genes and the presence of high-risk populations may help to explain the geographic differences in prolific CA-MRSA clones, and could at least partially account for the apparent ecological niche that seemingly encourages the transmission of some strains within a region, while limiting the spread of others.

Limitations of this retrospective, population-based study include the inability to account for changes in the healthcare provider behaviour over the 15-year study period, in particular the propensity to send specimens for culture and typing. It is reasonable to assert that a rapid rise of specific PVL+ isolates within a region, causing SSTIs in increasing number and severity, raised the profile of CA-MRSA among local clinicians and drove up testing numbers to an extent.

The observed increases in PVL− CA-MRSA observed mainly in the Kimberley region may, indeed, be largely driven by an increased propensity to test for CA-MRSA. Increased testing, however, should not have any impact on the proportion of PVL+ isolates comprising the total number of specimens, suggesting that the increase in PVL+ cases in regional WA is not entirely an artefact of increased testing, but instead represents a true outbreak within the state.

Despite this limitation, the increased rates of PVL− disease, combined with an almost 50% reduction in these clones proportionally demonstrates that PVL+ clones have not replaced PVL− clones, but rather added to the burden of disease within the state. While information on individual PVL− clones is not available, the relatively minor comparative increase in rates of these clones overall suggests that it is unlikely that one or more particular outbreak clones were responsible for driving this, as was observed with PVL+ clones.

An additional limitation was that Aboriginality was not available as an indicator for this dataset, prohibiting further investigation of this as a risk factor. In place of this variable, IRSAD was used as a proxy measure, to account for the relative socio-economic disadvantage prevalent in remote communities. After controlling for IRSAD in the regression model, there were still significantly increased proportions of CA-MRSA identified in the northern zones.

Public health management of this current outbreak remains a challenge. The suggested propensity for CA-MRSA, particularly PVL+ clones, to spread in hotter and more humid areas, particularly among vulnerable communities, suggests WA may expect similar increases in incidence rates across a number of regions in future. Countries with climatic conditions favourable for colonisation and spread of CA-MRSA should adopt proactive mitigation strategies, particularly in areas where vulnerable populations reside.

Further work to evaluate the impact of this outbreak on the healthcare system within affected regions could provide evidence of the increased burden on individual patients that results from severe SSTIs, and further quantify the economic impact to healthcare systems. Such evidence could be used as further justification for increased prevention efforts, both to halt the current outbreak and to prevent the emergence of virulent strains in future.
